# Why are clinical trials of deep brain stimulation terminated? An analysis of clinicaltrials.gov (new version)

**DOI:** 10.1016/j.bas.2024.104137

**Published:** 2024-11-13

**Authors:** Muhammad Daniyal Shafqat, Inibehe Ime Okon

**Affiliations:** Shifa College of Medicine, Islamabad, Pakistan; Benjamin S. Carson (Snr) College of Health and Medical Sciences, Babcock University, IIishanRemo, Ogun State, 121003, Nigeria; Department of Neurosurgery, Dell Medical School, University of Texas at Austin, TX, 78712, United States

Dear Editor,

We would like to commend the substantive content of the article, particularly its insight into the very challenging domain of DBS clinical trials. The article suggests that a staggering proportion of trials, approximately 1 in 5, end up being abandoned for a multitude of reasons. Despite the attention drawn to this issue, we believe there is an opportunity to further explore not only the reasons for trial termination but also actionable recommendations to enhance the efficiency and effectiveness of DBS trials. This is a crucial prospectus for optimizing research strategies and improving patient outcomes.

Firstly, Deep brain stimulation (DBS) is a surgical technique in which a neurostimulator device is implanted to send electrical signals to specific areas of the brain. These signals can be externally adjusted, offering customized treatment based on the patient's needs. Originally used as a treatment for movement disorders, DBS has been explored for various other conditions, such as obsessive-compulsive disorder, major depressive disorder, dementia, Gilles de la Tourette syndrome, anorexia nervosa, and addictions (Georgiev et al., 2021). With the expansion of applications for DBSs, rigorously designed and conducted clinical trials are crucial for providing the robust evidence needed to guide their future use (Harmsen et al., 2020). However, a significant proportion of these trials have never reached completion (Mishra et al., 2024). We explore why.

Regarding the “factors affecting trial success”, perhaps the major obstacle to trial success is the adequate recruitment of participants, for which several factors contribute: stringent eligibility criteria exclude many patients (Eitan et al., 2018); the invasive nature of the procedure deters potential participants due to concerns about associated risks; the availability of alternative therapies such as vagal nerve stimulation, ketamine infusions, and transcranial magnetic stimulation reduces willingness to try experimental procedures; low professional referrals from psychiatrists critical of surgical interventions for depression limit patient access (Filkowski et al., 2016); and geographical and logistical barriers such as travel difficulties and costs impede participation. Suppose we successfully address all preceding challenges and achieve sufficient patient recruitment; another issue emerges: patient withdrawal or fatality during the research. This risk is particularly pronounced among older patients and those with movement disorders, where depression and suicidal ideation may coexist, potentially leading to fatal outcomes. Additionally, complications such as brain abscesses, hemorrhages, and wound infections (Rasiah et al., 2023) can further hinder patient participation. Moreover, technical issues, such as hardware failure, misplaced leads (Okun and Vitek, 2004), and programming errors, may also prompt patients to withdraw due to suboptimal responses.

Funding is particularly important due to the high cost of the treatment itself. DBS surgery can cost approximately $65,000, and trials often require multiple surgeries for battery replacements, adding another $10,000 to $20,000 per patient within a few years (Rossi et al., 2017). These significant costs can limit the number of participants included in trials, interfering with statistical analysis and rendering the study incomplete or less effective.

The existence of concurrent trials poses a potential risk to the success of the current trial, not only because of competition for participant attention but also because the lengthy duration of trials may allow for the publication of results from other trials. These results could suggest that alternative modalities are effective, rendering the current trial ethically questionable. For instance, if a trial was to indicate that another stimulation target (such as the superolateral medial forebrain bundle or slMFB) is more efficacious, it would be unethical to persist with the nucleus accumbens for deep brain stimulation (DBS) of treatment-resistant bipolar disorder (DBS-BIPO).

Finally, logistical challenges are omnipresent in the realm of clinical trials, encompassing the coordination of multicenter trials, data management, and compliance with regulatory standards. These complexities often result in trial delays, escalated costs, and premature terminations.

We recommend raising awareness about DBS through public campaigns, including information sessions and advertisements, to attract more potential candidates. Highlighting patient success stories and emphasizing the scientific rigor of the trials can help mitigate fears and encourage self-referral, which is particularly popular in the case of depression-related DBS trials (Brown et al., 2019). Moreover, efforts should be made to expand referral networks. Early outreach to community psychiatrists, family physicians, patient advocacy groups, and safety education initiatives can enhance participant recruitment (Ramasubbu et al., 2021).

Expanding on the discussion of technical challenges, it is important to highlight the significance of a comprehensive DBS treatment team for improving patient outcomes. Ideally, patients should receive programming at the implanting institution to ensure continuity of care. However, limited access to programmers presents a barrier to this goal. Furthermore, a lack of engagement from some DBS surgeons in the programming and follow up process creates a critical disconnect between surgical intervention and its long-term effectiveness. To bridge this gap and optimize patient care, fostering close collaboration within a dedicated DBS treatment team is essential. This team, composed of neurologists, programmers, and surgeons, ensures seamless communication and data exchange. Surgeons receive crucial feedback on treatment outcomes, allowing them to refine their surgical techniques and make informed programming adjustments for each patient. This collaborative approach would not only improve patient outcomes but also provide valuable data for ongoing research and development of DBS therapies (Okun et al., 2005).

To address funding issues, clinical trials can diversify their funding sources by seeking grants from government agencies, private foundations, and industry partners. This reduces reliance on a single source and minimizes the risk of financial shortfalls. Collaborating with established institutions can also enhance credibility and attract additional investment. Ensuring rigorous peer review processes, similar to those used by the NIH, can improve study quality, increasing the likelihood of success and ongoing funding.

Various countries and organizations have developed registries to collect data on DBS procedures, outcomes, and patient demographics. A notable example is ClinicalTrials.gov, which, although perhaps the most prominent registry, has limitations. This information may be incomplete, especially for studies not labeled clinical trials, and is biased toward U.S.-based trials, potentially omitting important international data. We suggest a central registry for therapeutic DBS trials to enable key stakeholders, including investigators, clinicians, and regulators, to access trial-specific information, such as study design, outcomes, and adverse events, more effectively (Synofzik et al., 2012).

In conclusion, deep brain stimulation (DBS) holds promise for various conditions but faces challenges in clinical trials, including participant recruitment, patient withdrawal, funding, competition, and logistical issues. Improving recruitment through awareness campaigns and expanded referral networks, establishing comprehensive DBS treatment teams, and diversifying funding sources can address these obstacles. Additionally, creating a central registry for DBS trials will enhance access to crucial trial information for stakeholders. By implementing these strategies, we can overcome current barriers and fully realize the potential of DBS therapies.

## Authors’ contributions

All the authors (M.D.S., and I.I.O.) have made equal contributions.

## Funding statement

None.

## Declaration of competing interest

Not applicable.
